# Neuroinflammation is a susceptibility factor in developing a PTSD-like phenotype

**DOI:** 10.3389/fnbeh.2023.1112837

**Published:** 2023-03-29

**Authors:** Khadijah Shanazz, Rebecca Nalloor, Rudolf Lucas, Almira Vazdarjanova

**Affiliations:** ^1^VA Research Service, Charlie Norwood VA Medical Center, Augusta, GA, United States; ^2^Department of Pharmacology and Toxicology, Medical College of Georgia, Augusta University, Augusta, GA, United States; ^3^Vascular Biology Center, Medical College of Georgia, Augusta University, Augusta, GA, United States; ^4^Division of Pulmonary and Critical Care Medicine, Medical College of Georgia, Augusta University, Augusta, GA, United States

**Keywords:** IL-6, IL-10, anxiety, startle behavior, rats, susceptibility, TNFa, IL-1b

## Abstract

**Introduction:**

Post-Traumatic Stress Disorder (PTSD) is a psychological disorder that occurs after a traumatic event in a subset of exposed individuals. This implies the existence of susceptibility factors that foster the development of PTSD. Susceptibility factors are present before trauma and can contribute to the development and maintenance of PTSD after trauma. Manipulation of susceptibility factors may decrease the probability of developing PTSD. A putative susceptibility factor is inflammation. Patients with PTSD have been documented to have a higher pro-inflammatory profile compared to non-PTSD subjects. In addition, they are more likely to develop and die from cardiovascular disease which has a strong inflammation component. It is not known, however, whether inflammation plays a role in developing PTSD or whether reducing inflammation can prevent PTSD.

**Methods:**

We used the Revealing Individual Susceptibility to a PTSD-like phenotype (RISP) model to behaviorally classify male rats as resilient or susceptible before trauma and tested their serum and prefrontal cortical (mPFC) levels of IL-1β, IL-6, TNFα, IL-10, IFN IFNγ, and KC/GRO to determine whether inflammation represents a putative susceptibility factor for PTSD.

**Results:**

We found elevated IL-6 levels in the mPFC, but not serum, of susceptible rats compared to resilient animals before trauma. Serum and mPFC levels were not correlated in any of the cytokines/chemokines. Rats with high anxiety-like behavior had elevated IL-6 and IL-10 mPFC levels. Acoustic startle responses were not associated with cytokine/chemokine levels.

**Discussion:**

Neuroinflammation, rather than systemic inflammation exists in susceptible male rats before trauma and is thus a putative susceptibility factor for PTSD. Thus, susceptibility appears neurogenic in its pathogenesis. The lack of differences between susceptible and resilient rats in serum cytokine/chemokine levels infers that peripheral markers will not be useful in determining susceptibility. Chronic neuroinflammation appears more broadly associated with anxiety rather than startle responses.

## Graphical Abstract

Peripheral and neuroinflammatory profile in rats susceptible or resilient to a PTSD-like phenotype. 
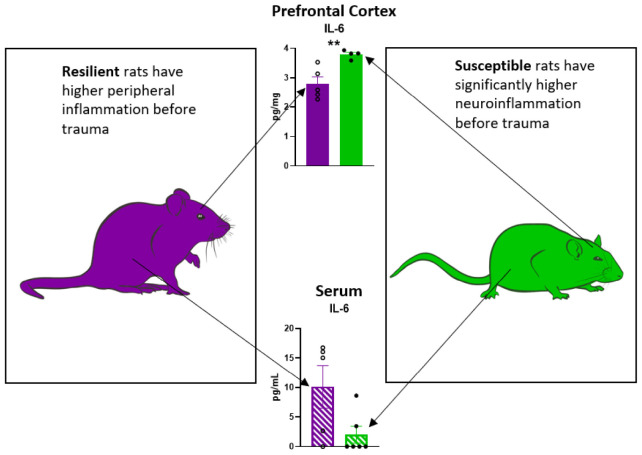

## 1. Background

Post-Traumatic Stress Disorder (PTSD) develops in a subset of people who experience a traumatic event with or without physical trauma. As a result of the trauma, patients with PTSD develop symptoms such as nightmares, intrusive memories, flashbacks, and disruptions in activities of daily living which persist for more than 30 days after trauma exposure (American Psychiatric Association, 2013). The presence of intrusive memories and flashbacks suggests that PTSD is, in part, a memory disorder and substantiates the view that it is the most severe of fear disorders (Sher and Vilens, 2010; Izquierdo et al., 2016). They also have a higher likelihood of developing cardiovascular disease (Edmondson and von Känel, 2017) and are at a significant risk of disability and suicide (Mitchell et al., 2021).

Since trauma is unpredictable and PTSD is difficult to treat (Sessa, 2017), it is medically important to identify susceptibility factors in order to improve therapeutic treatment regimens that can confer resilience against developing PTSD. We define susceptibility factors as conditions that exist before trauma and that increase the probability of developing and maintaining PTSD after trauma and which can be altered to change PTSD outcomes (Alexander et al., 2020). We have previously shown that animals susceptible to developing a PTSD-like phenotype have impaired hippocampal function prior to experiencing trauma (Nalloor et al., 2014) and have difficulty in learning safety (impaired extinction) after trauma (Nalloor et al., 2011) similar to what is seen in humans (Jovanovic et al., 2010, 2012). In addition, PTSD patients have pre-frontal cortical (PFC) dysfunction marked by dysregulation of emotion, inappropriate recall of fear memories, impaired learning of safety, and flashbacks of the traumatic experience (Gamwell et al., 2015; Hourani et al., 2015; Izquierdo et al., 2016; Gobin et al., 2017).

It is well documented that patients with established PTSD also have increased levels of circulating pro-inflammatory cytokines, such as TNFα, IL-1β, IFN-γ, and IL-6 (Passos et al., 2017; Speer et al., 2018; Wang et al., 2019). Inflammation may be involved in the maintenance of PTSD pathology as inflammatory markers between people with PTSD experiencing symptom remission and no-PTSD controls are similar (O’Donovan et al., 2017). Additionally, some studies report a chronic inflammation in people with PTSD (Hori and Kim, 2019), as evidenced by high levels of the anti-inflammatory cytokine IL-10 (a negative feedback mechanism toward an ongoing inflammation) and of the pro-inflammatory cytokine IL-6 (de Oliveira et al., 2018). However, in contrast to these studies, others have reported decreased circulating levels of pro-inflammatory cytokines and chemokines in people with PTSD, including IL-6 (Agorastos et al., 2019) and CXCL1 (Bam et al., 2016), respectively. Moreover, a peri-trauma study found that lower levels of peripheral TNF and IFN-γ were associated with a higher risk of persistent PTSD (Lalonde et al., 2021), suggesting that higher levels of these pro-inflammatory cytokines during trauma can be protective. It would be ideal, if the inflammatory state of the central nervous system (neuroinflammation) in PTSD patients could be inferred from the measurement of peripheral blood markers. However, despite of the fact that the majority of findings from recent studies using peripheral markers point toward a higher proinflammatory state in people with PTSD, a study using post-mortem brains of people with PTSD intriguingly found lower levels of IL-1α gene expression in the prefrontal cortex (PFC) compared to controls (Morrison et al., 2019). As such, in the absence of peripheral and central markers measured in the same subjects it is possible that peripheral markers may not be adequate to infer neuroinflammation in PTSD and rather local markers should be assessed. Taken together, these opposing findings from the different studies reported above document that inflammation in people with PTSD is highly complex. This stresses the need for more research investigating the role of systemic (peripheral) and local (neuro) inflammation in the pathogenesis and maintenance of PTSD.

Furthermore, it is not clear whether peripheral inflammation or neuroinflammation represent susceptibility factors. Few studies have examined inflammation prior to trauma exposure. A study in humans found that high levels of circulating C-reactive protein (CRP) prior to military deployment were associated with developing PTSD after combat exposure (Eraly et al., 2014) suggesting that inflammation is a potential susceptibility factor. Preclinical studies of inflammation and stress also support the idea that inflammation plays a role in susceptibility. Notably, it has been shown that early life inflammation induced by lipopolysaccharide (LPS) in rats is associated with impaired fear extinction at 2 months of age (Doenni et al., 2017). In mice, social defeat stress was associated with increased serum levels of TNFα and IL-6 (Azzinnari et al., 2014). However, the hypothesis that inflammation is a susceptibility factor has not been experimentally tested in the context of PTSD.

One available tool for examining susceptibility factors is the Revealing Individual Susceptibility to PTSD-like phenotype (RISP) model. The RISP model classifies male rats as susceptible (Sus), resilient (Res), or intermediate using behavioral tests of anxiety and startle 4 days after exposure to a mild stressor but before exposure to a traumatic experience (Nalloor et al., 2011; Alexander et al., 2020). The mild stressor in the RISP model is a necessary step for revealing susceptibility. Sus animals (∼20%) have high anxiety and startle responses and Res animals (∼30%) have low anxiety and startle responses. Res and Sus classifications correlate with a post-trauma PTSD-like phenotype such that Sus animals have impaired fear extinction and maintain or increase their startle response for at least 3 weeks after trauma (Nalloor et al., 2011; Alexander et al., 2020). In addition to behavioral differences between Sus and Res before trauma, Sus animals have impaired hippocampal function as measured by plasticity related immediate early genes *Arc* and *Homer1a* compared to Res (Nalloor et al., 2014). Thus, classification with the RISP model is predictive of post-trauma phenotype yet functional and phenotypical differences exist and can be identified before trauma.

To determine whether systemic or neuroinflammation represent susceptibility factors to developing PTSD, we measured pre-trauma levels of the pro-inflammatory cytokines IL-6, IL-1β, TNFα, and IFN-γ, the anti-inflammatory cytokine IL-10, and the pro-inflammatory neutrophil-attractant chemokine KC/GRO/CINC-1 in the serum and pre-frontal cortex of rats classified as Sus or Res according to the RISP model. We tested the hypothesis that Sus rats would have higher pro-inflammatory cytokines compared to Res. We predicted that there would be an elevation in at least one of the pro-inflammatory cytokines in either serum and/or PFC, no change or decrease in IL-10, and no change in KC/GRO/CINC-1 as we do not expect there to be involvement of the adaptive immune response.

## 2. Materials and methods

### 2.1. Animals and handling

A total of 24 young adult (2 months old) male (250–300 g) Sprague–Dawley rats (Charles River Laboratories Inc., Wilmington, MA, USA) were housed in pairs on a 12 h light/dark cycle (lights on at 7:00 a.m.) with food and water freely available. All rodents were handled for 2–3 min for three consecutive days, starting 3 days after arrival. Behavioral testing began after handling. Experiments were conducted during the light phase between 8 a.m. and 5 p.m. All procedures were approved by the Institutional Animal Care and Use Committee (IACUC) at the Charlie Norwood VA Medical Center (CNVAMC). The number of rats per group is listed in each figure.

### 2.2. Classification using the revealing individual susceptibility to PTSD-Like phenotype (RISP) model

**FIGURE 1:**
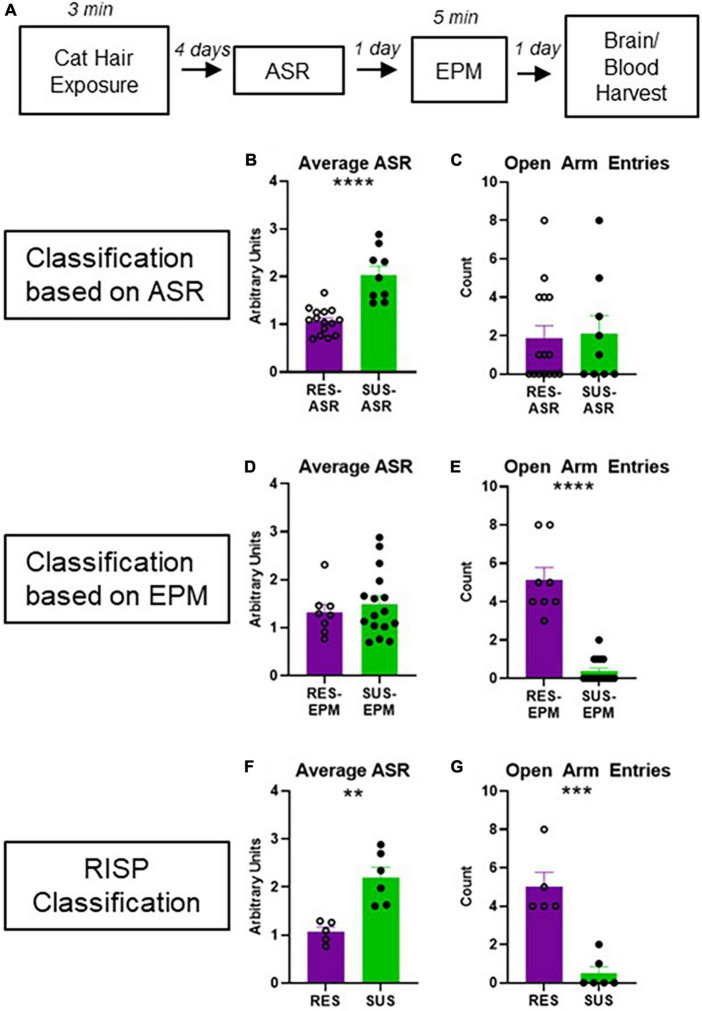
Experimental design and behavioral classification. **(A)** Experimental design; **(B)** average ASR of rats classified as Res-ASR or Sus-ASR (Res *n* = 15, Sus *n* = 9); **(C)** number of EPM Open Arm Entries of rats classified as Res-ASR or Sus-ASR (Res *n* = 15, Sus *n* = 9); **(D)** average ASR of rats classified as Res-EPM or Sus-EPM (Res *n* = 8, Sus *n* = 16); **(E)** number of EPM Open Arm Entries of rats classified as Res-EPM or Sus-EPM (Res *n* = 8, Sus *n* = 16); **(F)** average ASR of rats classified as Res or Sus with the RISP model (Res *n* = 5, Sus *n* = 6); **(G)** number of EPM Open Arm Entries of rats classified as Res or Sus with the RISP model (Res *n* = 5, Sus *n* = 6). **p < 0.01, ***p < 0.001, and ****p < 0.0001.

Animals were classified using the RISP model as previously described (Figure 1A; Nalloor et al., 2011; Alexander et al., 2020).

#### 2.2.1. Mild stressor (CH)

Rats are first exposed to a ball of cat hair infused with 150 ul of fresh cat urine, 10 cm in diameter, obtained from a pathogen free male cat. The cat hair was placed in one corner of a 35 cm × 26 cm × 50 cm box. The box was divided into four equal quadrants. Each animal was introduced into the quadrant furthest from the cat hair and allowed to explore the box freely for 3 min. The box was wiped clean between animals with dH_2_O. Cat hair exposure under these conditions is considered a mild stressor as they do not display freezing the next day when placed in an identical box in the absence of the cat hair (Nalloor et al., 2011).

#### 2.2.2. Acoustic startle response (ASR)

We measured their ASR 4 days after CH exposure as we have seen from previous experiments that this is sufficient time for the stress response elicited by the cat hair exposure to subside (Nalloor et al., 2011). Testing was performed in sound attenuated startle chambers (SR-LAB, San Diego Instruments, San Diego, CA, USA) with clear acrylic restraining tubes. Each animal was presented with fifteen 120 dB acoustic bursts (40 ms each), at random intervals (30–45 s). Acoustic startle response (ASR) was measured as the displacement of the restraining tube detected by a piezoelectric device at its base and reported in arbitrary units.

#### 2.2.3. Elevated plus maze (EPM)

We measured anxiety-like behavior on the EPM the day after ASR. The EPM is plus-shaped with four 50 cm × 12 cm arms, elevated 84 cm above the floor. Two opposite arms are surrounded by 46 cm tall opaque black walls on three sides, and the other two are open, except for a 1 cm high ledge (Kinder Scientific, San Diego, CA, USA). Each animal was introduced into the center area (10 cm × 10 cm) facing an Open Arm and allowed to explore freely for 5 min. Entries into the Open and Closed Arms were scored. An Arm Entry was scored when all four paws and the base of the animal’s tail entered an arm.

#### 2.2.4. Classification criteria

Acoustic startle response Classification–The animals were classified according to their ASR with the following criteria:

Susceptible (Sus-ASR)–Average ASR and 6 or more individual ASR were greater than the group average ASR.

Resilient (Res-ASR)–Average ASR and more than 7 individual ASR were smaller than the group average ASR.

EPM Classification–The animals were classified according to their EPM behavior with the following criteria:

Susceptible (Sus-EPM)–Fewer than 2 entries into the open arms (a behavior associated with high anxiety).

Resilient (Res-EPM)–At least 2 entries into the open arms.

RISP Classification–The animals were classified with both ASR and EPM criteria:

Susceptible (Sus)–Animals classified as Susceptible on both ASR and EPM.

Resilient (Res)–Animals classified as Resilient on both ASR and EPM.

Animals meeting neither set of criteria were excluded from RISP analysis.

Previous studies have shown that RISP Sus phenotype is associated with impaired fear extinction after experiencing foot shock and an elevated startle response 2 weeks after foot shock compared to Res animals (Nalloor et al., 2011).

### 2.3. Tissue sample collection and processing

The animals were sacrificed the day after EPM. Brains were harvested and trunk blood was collected immediately after sacrifice (Figure 1A). Trunk blood was collected in tubes and allowed to clot at room temperature for 30 min before being spun at 12,000 g in a refrigerated centrifuge for 20 min at 4°C. Serum samples were acquired from the supernatant, aliquoted, and stored at −80°C until cytokine measurement. Brains were rapidly extracted, flash frozen, and stored at −80°C. Left hemispheres from brains were sliced and PFC was dissected with a large tip flat needle. PFC was identified between bregma 2.52 and 3.72 mm using the shape of the forceps minor corpus callosum (fmi) as a visual guide. The tissue medial of the fmi was dissected carefully, avoiding the cingulate cortex just above. PFC tissue was homogenized in RIPA buffer (8,990, Thermo Scientific, Rockford, IL, USA) with protease inhibitor (P2714, Sigma Aldrich, St. Louis, MO, USA) and protein concentration was measured with a BCA assay kit (23,227, Thermo Scientific, Rockford, IL, USA) according to kit instructions using sample duplicates. Only homogenate samples with sufficient protein for cytokine quantification were used.

### 2.4. Cytokine measurement

Serum and PFC homogenate cytokine levels were measured using commercially available kits (K15059D, Meso Scale Discovery, Rockville, MD, USA). The 96-well V-Plex rat proinflammatory panel 2 was used for both serum and PFC homogenates with duplicate samples. The multiplex apparatus (MESO QuickPlex SQ 120, Meso Scale Discovery, Rockville, MD, USA) allowed for simultaneous measurement of IL-1β, IL-6, TNFα, IL-10, IFN-γ, and KC/GRO within each sample. Serum samples were run according to kit protocol instructions. PFC homogenates were run with the same protocol with the modification of an overnight sample incubation in a refrigerator to increase sensitivity.

### 2.5. Statistics

Unpaired *T*-tests were used to compare Res versus Sus animals. Kolmogorov–Smirnov test for normality was used on all data sets. Groups that did not pass normality were tested for differences using the non-parametric Mann–Whitney test (noted in the relevant section “3. Results”). Differences were considered statistically significant at *p* < 0.05 data was analyzed and visualized with PRISM (GraphPad Prism version 8.0.0 for Windows, GraphPad Software, San Diego, CA, USA).

## 3. Results

### 3.1. RISP classification is selective for behavioral differences

Acoustic startle response classification on its own (Figure 1B) does not reveal differences in anxiety-like behavior on the EPM (Figure 1C, *p* = 0.8267). Likewise, EPM classification on its own (Figure 1E) does not reveal differences in ASR (Figure 1D, *p* = 0.5288). As expected, using RISP classification, a combination of the two, shows differences on both ASR and EPM (Figures 1F, G, *p* = 0.0003 and *p* = 0.0019, respectively). Importantly, previous research has shown that animals classified with the RISP model show a complex post- trauma PTSD-like phenotype- they have both impaired fear extinction and long-lasting elevations in ASR which are trauma-dependent. In contrast, rats classified by the ASR or EPM classifications alone show singular aspects of this phenotype- Sus-ASR rats show only elevated long-lasting startle, while Sus-EPM rats show impaired extinction but not long-lasting elevation in startle compared to the Res-ASR and Res-EPM rats, respectively (Nalloor et al., 2011). Additionally, ROC analysis for RISP classification predictability of impaired extinction shows area under the curve (AUC) = 0.6368 and *p* < 0.05.

Using the RISP classification we tested the hypothesis that Sus animals have higher pre-trauma inflammation.

### 3.2. RISP classification reveals neuroinflammation in Sus rats before trauma

**FIGURE 2:**
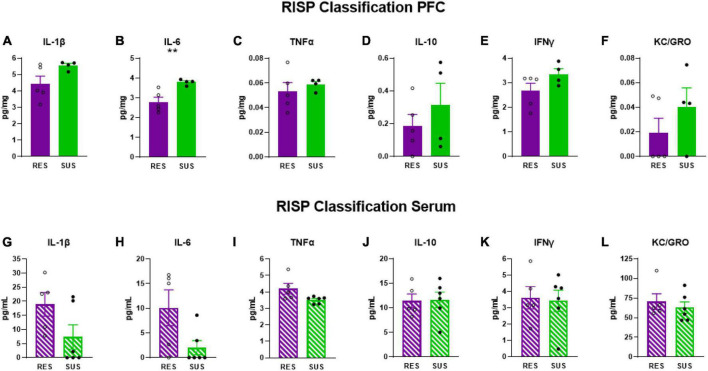
IL-1β, IL-6, TNFα, IL-10, IFNγ, and KC/GRO levels in PFC and serum based on RISP classification (PFC: Res *n* = 5, Sus *n* = 4, Serum: Res *n* = 5, Sus *n* = 6). Protein levels of IL-1β, IL-6, and TNFα are in pg/mg for PFC and in pg/mL for serum. **p < 0.01.

IL-6 levels were elevated in the PFC (Figure 2B *p* = 0.0085), but not in serum (Figure 2H), of Sus animals compared to Res. Additionally, IL-1β levels in the PFC of Sus animals were nearly significantly higher (Figure 2A *p* = 0.0798). Such findings support the hypothesis that, before trauma, neuroinflammation rather than peripheral inflammation is a susceptibility factor. There were no significant differences observed in PFC for levels of TNFα, IL-10, IFNγ, and KC/GRO between Res and Sus animals (Figures 2 C–F). Intriguingly, serum cytokine levels of IL-1β (Figure 2G, *p* = 0.089), IL-6 (Figure 2H, *p* = 0.053), and TNFα (Figure 2I, *p* = 0.055), tended to be higher in Res compared to Sus rats but this did not reach significance. Serum levels of IL-10, IFNγ, and KC/GRO were similar between Res and Sus rats (Figures 2J–L). Notably, we found no correlation between PFC and serum cytokine levels in Res or Sus rats (Table 1), suggesting that neuroinflammatory levels of cytokines cannot be inferred from serum levels under these conditions. We further examined if any differences could be revealed by ASR or EPM classifications alone.

**TABLE 1:** Correlations between PFC and serum in RISP classified res and Sus animals.

	Resilient	Susceptible
IL-1β	*R*^2^ = 0.270, *P* = 0.3700	*R*^2^ = 0.013, *P* = 0.8879
IL-6	*R*^2^ = 0.195, *P* = 0.4572	*R*^2^ = 0.028, *P* = 0.8336
TNFα	*R*^2^ = 0.005, *P* = 0.9083	*R*^2^ = 0.115, *P* = 0.6616
IL-10	*R*^2^ = 0.095, *P* = 0.6143	*R*^2^ = 0.697, *P* = 0.1650
IFNγ	*R*^2^ = 0.397, *P* = 0.2549	*R*^2^ = 0.473, *P* = 0.3123
KC/GRO	*R*^2^ = 0.167, *P* = 0.4941	*R*^2^ = 0.276, *P* = 0.4747

### 3.3. ASR classification does not reveal higher inflammation in Sus rats before trauma

**FIGURE 3:**
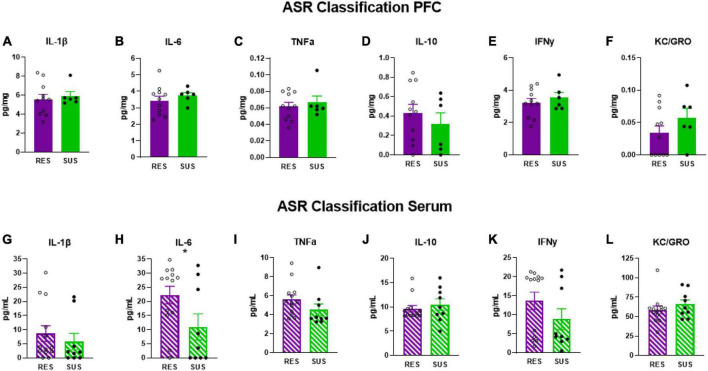
IL-1β, IL-6, TNFα, IL-10, IFNγ, and KC/GRO levels in PFC and serum based on ASR classification (PFC: Res *n* = 11, Sus *n* = 6, Serum: Res *n* = 13, Sus *n* = 9). Protein levels of IL-1β, IL-6, and TNFα are in pg/mg for PFC and in pg/mL for serum. *p < 0.05.

Susceptible-ASR rats have significantly lower serum levels of IL-6 (Figure 3H, *p* = 0.0475) suggesting that higher levels of serum IL-6 are associated with lower startle responses. No differences were detected in serum in any of the other cytokines measured (Figures 3G, I–L). No differences were detected in the PFC on any of the cytokines measured (Figures 3A–F).

### 3.4. EPM classification is associated with chronic neuroinflammation in Sus-EPM rats before trauma

**FIGURE 4:**
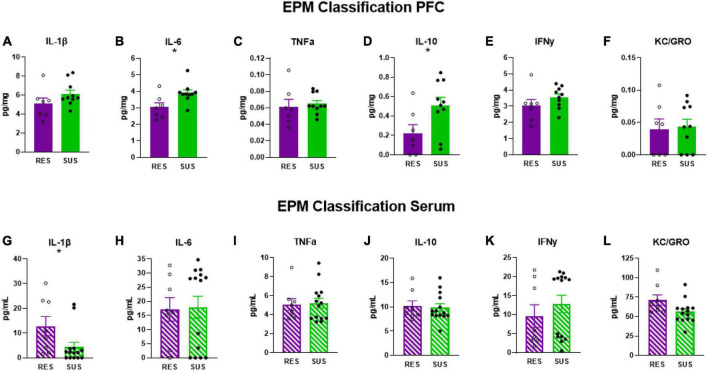
IL-1β, IL-6, TNFα, IL-10, IFNγ, and KC/GRO levels in PFC and serum based on EPM classification (PFC: Res *n* = 7, Sus *n* = 10, Serum: Res *n* = 8, Sus *n* = 14). Protein levels of IL-1β, IL-6, and TNFα are in pg/mg for PFC and in pg/mL for serum. *p < 0.05.

Susceptible-EPM rats have elevated levels of IL-6 (Figure 4B, *p* = 0.0158) and IL-10 (Figure 4D, *p* = 0.0352) in the PFC suggesting that higher anxiety is associated with chronic neuroinflammation. No other differences were detected in PFC (Figures 4A, C, E, F). Interestingly, IL-1β in the serum of Sus-EPM animals was decreased compared to Res-EPM (Figure 4G, *p* = 0.0233, U = 23, Mann–Whitney test used due to not normally distributed values). No other differences were detected in serum (Figures 4H–L).

## 4. Discussion

It is well-documented that PTSD is associated with inflammation, however, there is limited information on the role of inflammation in susceptibility to developing PTSD. Because PTSD is difficult to treat, it is important to understand what factors contribute to susceptibility to tailor interventions that can confer resilience. Here we examined cytokine and chemokine levels in animals classified as Susceptible (Sus) and Resilient (Res) using ASR, EPM, and the RISP model before trauma in serum and prefrontal cortex (PFC).

We used three different classification methods in male Sprague-Dawley rats because it is an outbred strain with a broad genetic background suitable for revealing individual differences in a PTSD-like phenotype. Although we present the data from all three classifications, we want to stress the data from the RISP model which was designed to incorporate multiple aspects of PTSD that can be modeled in rodents (anxiety, startle, impaired learning of safety) and classifies rats before trauma for susceptibility to a post-trauma PTSD-like phenotype. Thus, it models best the complex characteristics of PTSD. ASR or EPM classifications alone show singular aspects of this phenotype- Sus-ASR rats show only elevated long-lasting startle, while Sus-EPM rats show impaired extinction but not long-lasting elevation in startle compared to the Res-ASR and Res-EPM rats, respectively after trauma exposure (Nalloor et al., 2011; Alexander et al., 2020). Additionally, we have previously shown that animals identified as Sus with the RISP model have altered hippocampal function before trauma (Nalloor et al., 2014).

Our data demonstrate that Sus rats classified with the RISP model have significantly higher levels of IL-6 (Figure 2B) and nearly significantly higher levels of IL-1β in PFC (Figure 2A), but not serum, before trauma compared to Res rats which supports the hypothesis that neuroinflammation, rather than peripheral inflammation, is a susceptibility factor. We also found a tendency, albeit not significant, of higher serum levels of IL-1β, IL-6, and TNFα in Res rats compared to Sus before trauma (Figures 2G–I). This may be residual inflammation from the mild stressor exposure (cat hair) and/or ASR and EPM tests that are required in the RISP model for classification (Nalloor et al., 2011). Because the cat hair exposure is required to reveal susceptibility, in its absence virtually all animals behave as resilient, it is likely that the mild stressor (cat hair) is affecting Res rats with peripheral inflammation (Figure 2H), whereas Sus rats develop neuroinflammation (Figure 2B). Combined, the current findings suggest that the behavioral differences that emerge after cat hair exposure in the RISP classification are influenced by inflammatory status (Nalloor et al., 2011; Alexander et al., 2020). The results demonstrate that the role and location of inflammation in susceptibility to PTSD-like conditions can be different between susceptible and resilient animals.

Based on the RISP model, our data indicate that serum cytokine levels may not be sufficient for determining susceptibility to PTSD, as we found no correlation between serum and PFC cytokine levels in Res or Sus rats (Table 1). Others have also found inconsistent levels of cytokines and chemokines in brain and blood in rats (Zahr et al., 2010; Abramova et al., 2013). Because the blood-brain barrier (BBB) modulates cytokine transport in and out of the brain (Banks et al., 1995; Chen and Reichlin, 1999; Yarlagadda et al., 2009) it is not surprising to find that brain and blood levels of these markers are different or not correlated in young rats with presumably intact BBB. It is known that cytokines affect BBB integrity over time (Saija et al., 1995; Rochfort and Cummins, 2015) and therefore our findings do not contradict that people with established PTSD have, in most studies, higher levels of peripheral pro-inflammatory cytokines which may correlate with neuroinflammation. Coupled with the finding of significantly higher IL-6 in PFC of susceptible rats prior to trauma, this suggests that the role of inflammation in PTSD susceptibility is neurogenic in nature. This is an important consideration because it points to the potential difficulty in assessing susceptibility using peripheral markers in heathy individuals which is currently one of the least invasive and most widely used methods for acquiring biomarkers in humans.

Startle and anxiety are symptoms of PTSD (clusters D&E) (American Psychiatric Association, 2013) and in the RISP model they are collectively predictive of PTSD-like phenotype after trauma. However, there is merit in examining these symptoms individually as well. Anxiety disorders are prevalent and share a high comorbidity with PTSD (Spinhoven et al., 2014). High startle response is a symptom of anxiety, PTSD, and hypersensitivity disorders (Butler et al., 1990; Morgan et al., 1995; Grillon, 2002; Lucker, 2013). Examining ASR and EPM classification criteria individually also yielded interesting findings about PTSD-associated behaviors and their relationship with inflammation. In animals classified based on ASR alone, which only predicts long-lasting elevation in ASR in Sus-ASR rats, we found a significant increase in serum IL-6 in Res-ASR rats (Figure 3H) suggesting that higher levels of circulating IL-6 may be protective against high startle responses and may be worth investigating as a potential marker to track improvement in startle related disorders. Using EPM criteria alone showed that higher levels of circulating IL-1β are associated with lower anxiety-like behavior (Figure 4G). Combined, peripheral inflammation appears to be associated with resilience to a PTSD-like phenotype. EPM classification reveals that higher anxiety-like behavior is associated with chronic neuroinflammation as Sus-EPM rats have high levels of both IL-6 and IL-10 in PFC (Figures 4B, D). Using mice, others have found increased peripheral cytokine levels that were associated with high anxiety-like behavior after restraint stress (Elkhatib et al., 2020) and blocking IL-6 was associated with resilience to social defeat stress (Hodes et al., 2014) which highlights the importance of considering animal model, stressor type and when cytokines are measured in translational extrapolation.

Although the work presented here uses a model that can be used to study a post-trauma phenotype, it can also be used to study pre-trauma differences. However, both cannot be done in the same animals due to the nature of stress. Previous attempts to examine blood before and after trauma in the same animals revealed that blood draws are stressful and impact subsequent behavior skewing it toward Sus phenotype (0% classified as Res, unpublished observations).

The presented data reveal that the role of inflammation in susceptibility to a PTSD-like phenotype is complex. While peripheral inflammation may be protective, neuroinflammation in a brain region implicated in PTSD (PFC) is associated with susceptibility. These findings suggest that pre-trauma anti-inflammatory interventions that decrease neuroinflammation may reduce the likelihood of developing PTSD. Whole body interventions such as exercise and anti-inflammatory diet might be a better approach to reduce susceptibility than targeting individual cytokines or pathways as more information about the pleiotropic effects of individual cytokines emerges (Mocellin et al., 2003; Kishimoto, 2006; Gough and Myles, 2020). Such interventions have been shown to reduce inflammation (Tolkien et al., 2019; Metsios et al., 2020) and have effects on the brain (increased BDNF levels) (Firth et al., 2018).

Regardless, it is critical to apply interventions such as these prior to experiencing trauma or soon after because once the debilitating effects of PTSD occur it is tremendously more difficult to implement. Ongoing studies are evaluating anti-inflammatory interventions in the RISP model to determine how effective they might be in conferring resilience. Additionally, we are currently testing if increasing inflammation will worsen susceptibility. The RISP model was initially developed in male rats and studies in our lab attempting to apply the model to female rats revealed that is it not predictive for females. Female rats display different anxiety-like behavior and startle is not predictive of their post-trauma phenotype (Shanazz et al., 2022). Therefore, a new model for predicting a PTSD-like phenotype in females needs to be developed before this question can be answered in females with tools that account for sexual dimorphism in anxiety and fear responses (Shanazz et al., 2022).

## Data availability statement

The original contributions presented in this study are included in the article/supplementary material, further inquiries can be directed to the corresponding author.

## Ethics statement

This animal study was reviewed and approved by the Charlie Norwood VA Medical Center IACUC.

## Author contributions

KS wrote the manuscript, collected the data, analyzed the data, and helped with experimental design. RN helped with writing, analysis, and experimental design. RL helped with experimental design and editing the manuscript. AV helped with writing manuscript, analyzed the data, designed the experiment, and provided funding. All authors contributed to the article and approved the submitted version.
